# A list of oribatid mites (Acari, Oribatida) of Vietnam

**DOI:** 10.3897/zookeys.546.6188

**Published:** 2015-12-16

**Authors:** Sergey G. Ermilov

**Affiliations:** 1 Tyumen State University, Tyumen, Russia

**Keywords:** Oribatid mites, species list, fauna, Vietnam

## Abstract

A species list of identified oribatid mite taxa (Acari, Oribatida) in the fauna of Vietnam is provided. During 1967–2015, a total of 535 species/subspecies from 222 genera and 81 families was registered. Of these, 194 species/subspecies were described as new for science from Vietnam.

## Introduction

The first data on oribatid mite fauna (Acari, Oribatida) of Vietnam were presented by Balogh and Mahunka (1967). Subsequent studies of oribatids during the next 40 years were fragmentary in character, and the main papers on descriptions of new taxa and new records are the following: Rajski and Szudrowicz (1974), Golosova (1983, 1984), Vu et al. (1985, 1987), Jeleva and Vu (1987), Mahunka (1987, 1988, 1989), Niedbała (1989, 2000, 2004), Vu (1990, 1993, 1994), Starý (1993), Vu and Thi (1995), Krivolutsky (1998), Vu and Nguyen (2000, 2005), and Pavlichenko (2001).

Until 2007, only 195 species were recorded in Vietnam. Vu (2007) summarized this data, but his paper omitted more than 50 species, and did not consider synonyms and modern systematics.

A significant increase on oribatid taxonomic knowledge for Vietnam was contributed by the author and colleagues, which is based on material collected during joint Russian-Vietnamese expeditions in 2006–2014: Ermilov and Anichkin (2010, 2011a-k, 2012a-d, 2013a-g, 2014a-g), Ermilov (2011, 2013), Ermilov et al. (2011a-c, 2012a-f, 2013a-c, 2014a-f), Niedbała and Ermilov (2013, 2014), Anichkin et al. (2014). At present, Vietnamese oribatid mites have been actively studied by several researchers (Ermilov 2015a, b; Ermilov and Anichkin 2015a, b; Ermilov and Bayartogtokh 2015; Ermilov and Corpuz-Raros 2015; Fernandez et al. 2015a, b; Vu et al. 2015).

The objective of this paper is to list all identified oribatid mite taxa known from Vietnam to date (1 September 2015), to present primary references (in square brackets) on descriptions of new species or new findings for each species, and to provide the subsequent faunistic and taxonomic studies.

General taxonomic system of Oribatida used in this paper mostly follows that of Subías (2004, online version 2015), Weigmann (2006), Norton and Behan-Pelletier (2009), and Schatz et al. (2011).

## Species list

**Acaronychidae**

*Loftacarus siefi* Lee, 1981 [Vu et al. 2014]

*Stomacarus ciliosus* Luxton, 1982 [Vu et al. 2014]

*Stomacarus leei* Mahunka, 1989 [Vu et al. 2014]

**Ctenacaridae**

*Ctenacarus araneola* (Grandjean, 1932) [Ermilov and Anichkin 2013e]

**Cosmochthoniidae**

Cosmochthonius (Cosmochthonius) lanatus (Michael, 1885) [Vu et al. 1985]

**Sphaerochthoniidae**

*Sphaerochthonius splendidus* (Berlese, 1904) [Ermilov et al. 2012c]

**Hypochthoniidae**

Eohypochthonius (Eohypochthonius) crassisetiger Aoki, 1959 [Ermilov et al. 2012c]

Eohypochthonius (Eohypochthonius) gracilis Jacot, 1936 [Nguyen and Vu 2012]

*Malacoangelia remigera* Berlese, 1913 [Ermilov et al. 2012b]

**Lohmanniidae**

*Annectacarus unilateralis* Hammer, 1973 [Ermilov and Anichkin 2015a]

*Bedoslomannia anneae* Fernandez, Theron, Rolland & Castillo, 2015 [Fernandez et al. 2015a]

*Haplacarus pairathi* Aoki, 1965 [Nguyen and Vu 2012]

Javacarus (Javacarus) kuehnelti Balogh, 1961 [Vu et al. 1985]

Lohmannia (Lohmannia) javana Balogh, 1961 [Vu et al. 1985]

Lohmannia (Carolohmannia) monosetosa Ermilov & Anichkin, 2014 [Ermilov and Anichkin 2014d]

*Meristacarus africanus annobonensis* Pérez-Íñigo, 1969 [Ermilov and Anichkin 2015a]

*Meristacarus madagascarensis obscurus* Aoki, 1965 [Golosova 1983]

*Meristacarus sundensis* Hammer, 1979 [Ermilov and Anichkin 2011i]

Mixacarus (Mixacarus) exilis Aoki, 1970 [Ermilov and Anichkin 2011i]

Mixacarus (Mixacarus) foliifer Golosova, 1984 [Golosova 1984]

*Paillacarus aciculatus* (Berlese, 1908) [Vu et al. 1987]

*Paillacarus benenensis* Vu, Ermilov & Dao, 2010 [Vu et al. 2010]

*Paillacarus cornutus* Sarkar & Subías, 1984 [Ermilov and Anichkin 2011i]

*Paillacarus gueyeae* (Pérez-Íñigo, 1989) [Nguyen and Vu 2012]

*Papillacarus hirsutus* (Aoki, 1961) (=*Papillacarus arboriseta* Jeleva & Vu, 1987 – Ermilov et al. 2011a) [Golosova 1983]

*Paillacarus indistinctus* Ermilov, Anichkin & Wu, 2012 [Ermilov et al. 2012b]

*Paillacarus luteus* Ermilov, 2015 [Ermilov 2015b]

*Paillacarus polygonatus* Ermilov & Anichkin, 2011 [Ermilov and Anichkin 2011i]

*Paillacarus polysetosus* Ermilov, Anichkin & Wu, 2012 [Ermilov et al. 2012b]

*Paillacarus ramosus* Balogh, 1961 [Ermilov and Anichkin 2011i]

*Paillacarus undirostratus* Aoki, 1965 [Vu and Nguyen 2000]

*Papillacarus whitteni* Fernandez, Theron, Rolland & Leiva, 2015 [Fernandez et al. 2015b]

Paulianacarus (Paulianacarus) vietnamese Fernandez, Theron, Rolland & Castillo, 2015 [Fernandez et al. 2015a]

**Nehypochthoniidae**

*Nehypochthonius porosus* Norton & Metz, 1980 [Ermilov and Anichkin 2013e]

**Epilohmanniidae**

*Epilohmannia crassisetosa* Ermilov & Anichkin, 2012 [Ermilov and Anichkin 2012b]

*Epilohmannia cylindrica* (Berlese, 1904) [Vu et al. 1985]

*Epilohmannia dimorpha* Wallwork, 1962 [Nguyen and Vu 2012]

*Epilohmannia minuta pacifica* Aoki, 1965 [Ermilov et al. 2012c]

*Epilohmannia ornata* Mahunka, 1993 [Vu et al. 2014]

*Epilohmannia spathulata* Aoki, 1970 [Golosova 1983]

*Epilohmannoides xena* (Mahunka, 1983) [Vu et al. 2012]

**Protoplophoridae**

*Arthrhoplophora vulpes* Berlese, 1916 [Ermilov et al. 2012c]

**Mesoplophoridae**

*Apoplophora minuscula* Niedbała, 2013 [Niedbała and Ermilov 2013]

*Apoplophora pantotrema* (Berlese, 1913) [Golosova 1983]

**Oribotritiidae**

*Austrotritia lebronneci* (Jacot, 1934) [Niedbała and Ermilov 2014]

*Austrotritia saraburensis* Aoki, 1965 [Niedbała 2004]

*Indotritia javensis* Sellnick, 1923 [as *Indotritia completa* Mahunka, 1987 – Mahunka 1987]

*Oribotritia bulbifer* (Mahunka, 1987) [Ermilov et al. 2012c]

*Oribotritia paraaokii* Niedbała, 2000 [Niedbała 2000]

*Oribotritia submolesta* Niedbała, 2000 [Niedbała 2000]

**Euphthiracaridae**

*Acrotritia aokii* (Niedbała, 2000) [Niedbała 2000]

*Acrotritia ardua* (Koch, 1841) [Vu et al. 1985]

*Acrotritia duplicata* (Grandjean, 1953) [Vu et al. 1985]

*Acrotritia hyeroglyphica* (Berlese, 1916) [as *Rhysotritia hauseri* Mahunka, 1991 – Vu et al. 2012]

*Acrotritia otaheitensis* (Hammer, 1972) [Starý 1993]

*Acrotritia paragranulata* Niedbała, 2014 [Niedbała and Ermilov 2014]

*Acrotritia proxima* Niedbała, 2013 [Niedbała and Ermilov 2013]

*Acrotritia sinensis* Jacot, 1923 [as *Rhysotritia rasile* Mahunka, 1982 – Vu et al. 1987]

*Acrotritia vestita* (Berlese, 1913) [as *Rhysotritia comteae* Mahunka, 1983 – Niedbała 2000]

Euphthiracarus (Euphthiracarus) foveolatus Aoki, 1980 [Niedbała 2004]

Euphthiracarus (Euphthiracarus) labyrinthicus Starý, 1993 [Starý 1993]

Euphthiracarus (Euphthiracarus) quasitakahashii Niedbała, 2014 [Niedbała and Ermilov 2014]

Euphthiracarus (Euphthiracarus) parareticulatus Niedbała, 2000 [Niedbała 2000]

Euphthiracarus (Euphthiracarus) vietnamicus Starý, 1993 [Starý 1993]

Euphthiracarus (Parapocsia) medius Niedbała, 2014 [Niedbała and Ermilov 2014]

*Mesotritia maerkeli* Sheals, 1965 [Niedbała and Ermilov 2014]

*Mesotritia spinosa* Aoki, 1980 [Starý 1993]

*Microtritia minima* (Berlese, 1904) [Niedbała 2000]

*Microtritia tropica* Märkel, 1964 [Starý 1993]

**Synichotritiidae**

*Sabahtritia dongnaiensis* Niedbała, 2013 [Niedbała and Ermilov 2013]

**Steganacaridae**

*Arphthiracarus parasentus* Niedbała, 2000 [Niedbała 2000]

*Arphthiracarus tubulus* (Hammer, 1972) [Ermilov et al. 2012c]

Atropacarus (Atropacarus) phyllophorus (Berlese, 1904) [Vu et al. 1985]

Atropacarus (Atropacarus) striculus (Koch, 1935) [Niedbała 2000]

Atropacarus (Hoplophorella) cucullatus (Ewing, 1909) [Ermilov et al. 2012c]

Atropacarus (Hoplophorella) hamatus (Ewing, 1909) [as *Hoplophorella cuneiseta* Mahunka, 1988 – Mahunka 1988; also as *Atropacarus floridae* Jacot, 1933 – Niedbała 2000]

Atropacarus (Hoplophorella) stilifer (Hammer, 1961) [Niedbała 2000]

Atropacarus (Hoplophorella) vitrinus (Berlese, 1913) [Golosova 1983]

*Austrophthiracarus evexus* (Niedbała, 2000) [Niedbała 2000]

*Austrophthiracarus pullus* (Niedbała, 1989) [Ermilov et al. 2012c]

*Austrophthiracarus sentus* Niedbała, 1989 [Niedbała 1989]

*Plonaphacarus kugohi* (Aoki, 1959) [Niedbała 2000]

*Plonaphacarus insignitus* Niedbała, 1989 [Niedbała 1989]

*Plonaphacarus scrupeus* Niedbała, 1989 [Niedbała 1989]

*Protophthiracarus finitima* Niedbała, 2002 [Vu 2013]

Steganacarus (Rhacaplacarus) spinus Niedbała, 2014 [Niedbała and Ermilov 2014]

**Phthiracaridae**

*Hoplophthiracarus clavatus* Niedbała, 2014 [Niedbała and Ermilov 2014]

*Hoplophthiracarus pakistanensis* Hammer, 1977 [Nguyen and Vu 2012]

*Hoplophthiracarus stigmosus* Niedbała, 2000 [Niedbała 2000]

*Notophthiracarus nitidus* (Pérez-Íñigo & Baggio, 1988) [Nguyen and Vu 2012]

*Notophthiracarus perparvus* Niedbała, 1989 [Niedbała 1989]

*Phthiracarus abstemius* Niedbała, 1989 [Niedbała 1989]

*Phthiracarus crispus* Hammer, 1972 [Niedbała 2000]

*Phthiracarus invenustus* Niedbała, 2000 [Niedbała 2000]

*Phthiracarus paucus* Niedbała, 1991 [Niedbała 2000]

*Phthiracarus pygmaeus* Balogh, 1958 [Niedbała 2000]

**Trhypochthoniidae**

*Afronothrus incisivus* Wallwork, 1961 [Ermilov et al. 2012a]

*Allonothrus russeolus* Wallwork, 1960 [Nguyen and Vu 2012]

*Archegozetes longisetosus* Aoki, 1965 [Vu 1990]

**Malaconothridae**

*Malaconothrus dorsofoveolatus* Hammer, 1979 [Ermilov et al. 2012c]

*Tyrphonothrus adilatatus* (Ermilov, Anichkin & Tolstikov, 2014) [Ermilov et al. 2014a]

*Tyrphonothrus angustirostrum* (Hammer, 1966) [Nguyen and Vu 2012]

*Tyrphonothrus geminus* (Hammer, 1972) [Ermilov et al. 2012c]

*Tyrphonothrus variosetosus* (Hammer, 1971) [Ermilov et al. 2012b]

**Nothridae**

*Nothrus baviensis* Krivolutsky, 1998 [Krivolutsky 1998]

*Nothrus gracilis* Hammer, 1961 [Vu et al. 2012]

*Nothrus montanus* Krivolutsky, 1998 [Krivolutsky 1998]

*Nothrus oceanicus* Sellnick, 1959 [Vu et al. 2014]

*Nothrus shapensis* Krivolutsky, 1998 [Krivolutsky 1998]

**Crotoniidae**

*Heminothrus apophysiger* Hammer, 1969 [Ermilov and Anichkin 2014d]

**Nanhermanniidae**

*Cosmohermannia robusta* (Aoki, 1994) [Ermilov and Anichkin 2011i]

*Cyrthermannia vicinicornuta* Aoki, 1965 [Ermilov and Anichkin 2011i]

*Masthermannia mammillaris* (Berlese, 1904) [Ermilov and Anichkin 2011i]

*Nanhermannia thainensis* Aoki, 1965 [Vu 1993]

**Hermanniidae**

*Phyllhermannia bimaculata* Hammer, 1979 [Ermilov and Anichkin 2014f]

*Phyllhermannia forsteri* Balogh, 1985 [Vu et al. 2014]

*Phyllhermannia gladiata* Aoki, 1965 [Vu and Nguyen 2005]

*Phyllhermannia javensis* Hammer, 1979 [Nguyen and Vu 2012]

*Phyllhermannia similis* Balogh & Mahunka, 1967 [Balogh and Mahunka 1967]

**Hermanniellidae**

*Hermanniella bugiamapensis* Ermilov & Bayartogtokh, 2015 [Ermilov and Bayartogtokh 2015]

*Hermanniella aristosa* Aoki, 1965 [Ermilov and Anichkin 2014d]

*Hermanniella orbiculata* Hammer, 1979 [Vu et al. 2014]

*Hermanniella thani* Mahunka, 1987 [Mahunka 1987]

**Plasmobatidae**

*Plasmobates asiaticus* Aoki, 1973 [Ermilov and Anichkin 2014d]

**Neoliodidae**

*Neoliodes theleproctus* (Hermann, 1804) [Vu et al. 1985]

*Platyliodes japonicus* Aoki, 1979 [Nguyen and Vu 2012]

**Pheroliodidae**

*Pheroliodes longiceps* Balogh & Mahunka, 1966 [Vu et al. 2014]

**Licnodamaeidae**

*Hexachaetoniella dispersa* (Balogh, 1985) [Nguyen and Vu 2012]

*Pedrocortesella dongnaiensis* Ermilov & Anichkin, 2014 [Ermilov and Anichkin 2014f]

*Pedrocortesella pulchra* Hammer, 1961 [Nguyen and Vu 2012]

*Pedrocortesella temperata* Balogh, 1985 [Nguyen and Vu 2012]

*Pedrocortesella vietnamica* Ermilov & Anichkin, 2014 [Ermilov and Anichkin 2014f]

**Gymnodamaeidae**

*Arthrodamaeus vietnamicus* Ermilov & Anichkin, 2011 [Ermilov and Anichkin 2011b]

*Gymnodamaeus adpressus* (Aoki & Fujikawa, 1971) [Vu et al. 2008]

**Damaeidae**

*Belba corynopus* (Hermann, 1804) [Vu 1993]

*Metabelba orientalis* Balogh & Mahunka, 1967 [Balogh and Mahunka 1967]

*Tectodamaeus heterotrichus* Ermilov & Anichkin, 2014 [Ermilov and Anichkin 2014d]

**Cepheidae**

*Sphodrocepheus tuberculatus* Mahunka, 1988 [Mahunka 1988]

**Astegistidae**

*Cultroribula bicuspidata* Mahunka, 1978 [Ermilov et al. 2012c]

*Cultroribula lata* Aoki, 1961 [Golosova 1983]

Furcoppia (Furcoppia) cattienica Ermilov & Anichkin, 2012 [Ermilov and Anichkin 2012b]

Furcoppia (Furcoppia) parva Balogh & Mahunka, 1967 [Balogh and Mahunka 1967]

**Peloppiidae**

*Austroceratoppia japonica* Aoki, 1984 [Ermilov and Anichkin 2014d]

*Ceratoppia bipilis* (Hermann, 1804) [Ermilov and Anichkin 2014d]

*Ceratoppia crassiseta* Balogh & Mahunka, 1967 [Balogh and Mahunka 1967]

**Gustaviidae**

*Gustavia longicornis* (Berlese, 1904) [Ermilov and Anichkin 2014d]

**Zetorchestidae**

*Zetorchestes equestris* Berlese, 1908 [Golosova 1993]

*Zetorchestes saltator* Oudemans, 1915 [Vu 1990]

*Zetorchestes transvaalensis* Coetzee, 1989 [Nguyen and Vu 2012]

*Zetorchestes phylliferus* Mahunka, 1983 [Vu et al. 2014]

**Ctenobelbidae**

Ctenobelba (Berndamerus) bugiamapensis Ermilov, Shtanchaeva, Subías & Anichkin, 2014 [Ermilov et al. 2014f]

**Amerobelbidae**

*Roynortonia vietnamica* Ermilov, 2011 [Ermilov 2011]

**Eremulidae**

*Austroeremulus glabrus* Mahunka, 1985 [Nguyen and Vu 2012]

*Eremulus avenifer* Berlese, 1913 [Balogh and Mahunka 1967]

*Eremulus flagellifer* Berlese, 1908 [Nguyen and Vu 2012]

*Eremulus spinosus* Ermilov & Anichkin, 2011 [Ermilov and Anichkin 2011d]

*Eremulus truncatus* Hammer, 1971 [Golosova 1983]

*Mahunkana bifurcata* (Mahunka, 1987) [Mahunka 1987]

*Mahunkana japonica* (Aoki & Karasawa, 2007) [Ermilov et al. 2012c]

*Reteremuloides bifurcatus* Mahunka, 1989 [Ermilov and Bayartogtokh 2015]

**Damaeolidae**

*Fosseremus laciniatus* (Berlese, 1905) [Ermilov et al. 2012c]

*Gressittolus marginatus* Balogh, 1970 [Ermilov et al. 2012c]

**Hungarobelbidae**

*Costeremus ornatus* Aoki, 1970 [Vu et al. 2014]

**Eremobelbidae**

*Eremobelba bella* Hammer, 1982 [Nguyen and Vu 2012]

*Eremobelba bellicosa* Balogh & Mahunka, 1967 [Balogh and Mahunka 1967]

*Eremobelba breviseta* Balogh, 1968 [Ermilov et al. 2012c]

*Eremobelba capitata* Berlese, 1913 [Vu et al. 1985]

*Eremobelba hamata* Hammer, 1961 [Nguyen and Vu 2012]

*Eremobelba japonica* Aoki, 1959 [Vu et al. 2011]

**Heterobelbidae**

*Heterobelba stellifera stellifera* Okayama, 1980 [Ermilov et al. 2012a]

*Heterobelba stellifera formosana* Aoki, 1990 [Vu et al. 2014]

**Basilobelbidae**

*Basilobelba maidililae* Fernandez, Theron, Rolland & Leiva, 2015 [Fernandez et al. 2015b]

*Basilobelba parmata* Okayama, 1980 [Ermilov et al. 2012b]

*Basilobelba retiaria* (Warburton, 1912) [Ermilov and Anichkin 2013g]

*Xyphobelba hamanni* Csiszár, 1961 [Ermilov and Anichkin 2014d]

**Platyameridae**

*Gymnodampia crassisetiger* (Aoki, 1984) [Ermilov and Anichkin 2014d]

**Caleremaeidae**

*Epieremulus bidupensis* Ermilov & Anichkin, 2014 [Ermilov and Anichkin 2014d]

**Eremellidae**

*Eremella vestita* Berlese, 1913 [Vu and Nguyen 2000]

**Arceremaeidae**

*Tecteremaeus incompletus* Mahunka, 1988 [Ermilov and Bayartogtokh 2015]

*Tecteremaeus hauseri* Mahunka, 1982 [Ermilov et al. 2012c]

**Oppiidae**

*Acroppia processigera* (Balogh & Mahunka, 1967) [Nguyen and Vu 2012]

*Arcoppia arcualis* (Berlese, 1913) [Balogh and Mahunka 1967]

*Arcoppia corniculifera* (Mahunka, 1978) [Nguyen and Vu 2012]

*Arcoppia hammerae* Rodríguez & Subías, 1984 [Vu and Nguyen 2005]

*Arcoppia incerta* Balogh & Balogh, 1983 [Vu et al. 2014]

*Arcoppia longisetosa* Balogh, 1962 [Vu 1990]

*Arcoppia robustia* (Berlese, 1913) [Balogh and Mahunka 1967]

*Arcoppia serrutala* (Balogh & Mahunka, 1980) [Ermilov and Bayartogtokh 2015]

*Arcoppia viperea* (Aoki, 1959) [as *Arcoppia baloghi* Rodríguez & Subías, 1984 – Vu and Nguyen 2005]

*Arcoppia waterhousei* Balogh & Balogh, 1983 [Nguyen and Vu 2012]

*Belloppia shealsi* Hammer, 1968 [Vu et al. 2012]

Berniniella (Berniniella) bicarinata (Paoli, 1908) [Vu et al. 1985]

Brachioppiella (Brachioppiella) biseriata (Balogh & Mahunka, 1975) [Vu et al. 2012]

*Congoppia deboissezoni* (Balogh & Mahunka, 1966) [Vu et al. 2012]

*Cryptoppia elongata* Csiszár, 1961 [Vu et al. 1985]

*Cycloppia restata* (Aoki, 1963) [Ermilov and Anichkin 2013f]

Discoppia (Cylindroppia) cylindrica (Pérez-Íñigo, 1965) [Ermilov et al. 2012b]

*Elaphoppia quadripilosa* (Balogh, 1961) [Ermilov and Anichkin 2014d]

*Helioppia sol* (Balogh, 1959) [Vu et al. 2012]

Karenella (Karenella) acuta (Csiszár, 1961) [Vu et al. 1985]

*Kokoppia dendricola* (Jeleva & Vu, 1987) [Jeleva and Vu 1987]

Lanceoppia (Lancelalmoppia) becki Hammer, 1968 [Nguyen and Vu 2012]

Lanceoppia (Lanceoppia) translucens (Mahunka, 1985) [Vu et al. 2014]

Lasiobelba (Lasiobelba) kuehnelti (Csiszár, 1961) [Vu et al. 1985]

Lasiobelba (Lasiobelba) remota Aoki, 1959 [Balogh and Mahunka 1967]

Lasiobelba (Lasiobelba) vietnamica (Balogh, 1983) [as *Oppia remota* Aoki, 1959 – Balogh and Mahunka 1967]

*Lineoppia microseta* Ermilov & Anichkin, 2011 [Ermilov and Anichkin 2011g]

*Lyroppia dongnaiensis* Ermilov & Anichkin, 2013 [Ermilov and Anichkin 2013f]

*Microppia minus* (Paoli, 1908) [Ermilov et al. 2012b]

Multioppia (Multioppia) pseudoglabra Ermilov, 2015 [Ermilov 2015b]

Multioppia (Multioppia) tamdao Mahunka, 1988 [Mahunka 1988]

*Multipulchroppia pectinata* (Balogh & Mahunka, 1967) [Balogh and Mahunka 1967]

*Multipulchroppia similis* (Hammer, 1979) [Nguyen and Vu 2012]

Neoamerioppia (Neoamerioppia) vietnamica (Mahunka, 1988) [Mahunka 1988]

Oppiella (Oppiella) nova (Oudemans, 1902) [Golosova 1983]

*Oxybrachioppia barbata* (Choi, 1986) [Ermilov et al. 2012c]

Oxyoppia (Aciculoppa) clavata (Aoki, 1983) [Vu et al. 2008]

*Pulchroppia elegans* Hammer, 1979 [Ermilov et al. 2012c]

*Pulchroppia granulata* Mahunka, 1988 [Mahunka 1988]

*Pulchroppia roynortoni* Ermilov & Anichkin, 2011 [Ermilov and Anichkin 2011g]

Ramusella (Ramusella) chulumaniensis (Hammer, 1958) [Ermilov et al. 2012b]

Ramusella (Ramusella) clavipectinata (Michael, 1885) [Vu et al. 1985]

Ramusella (Ramusella) pocsi Balogh & Mahunka, 1967 [Balogh and Mahunka 1967]

Ramusella (Insculptoppia) elliptica (Berlese, 1908) [Ermilov et al. 2012c]

Ramusella (Insculptoppia) insculpta (Paoli, 1908) [Vu 1993]

*Ramuselloppia vietnamica* Ermilov & Anichkin, 2013 [Ermilov and Anichkin 2013b]

*Striatoppia lanceolata* Hammer, 1972 [Ermilov et al. 2013a]

*Striatoppia madagascarensis* Balogh, 1961 [Vu et al. 2012]

*Striatoppia opuntiseta* Balogh & Mahunka, 1968 [Mahunka 1988]

*Striatoppia papillata* Balogh & Mahunka, 1966 [Vu et al. 1985]

Taiwanoppia (Taiwanoppia) hungarorum (Mahunka, 1988) [Mahunka 1988]

**Granuloppiidae**

*Gigantoppia zryanini* Ermilov & Anichkin, 2011 [Ermilov and Anichkin 2011e]

*Granuloppia kamerunensis* Mahunka, 1974 [Nguyen and Vu 2012]

*Granuloppia vietnamensis* Ermilov & Bayartogtokh, 2015 [Ermilov and Bayartogtokh 2015]

Hammerella (Parawoasella) bayartogtokhi Ermilov, Shtanchaeva, Subías & Anichkin, 2012 [Ermilov et al. 2012f]

**Machuellidae**

*Machuella lineata* Hammer, 1973 [Ermilov et al. 2012b]

**Suctobelbidae**

*Allosuctobelba vietnamensis* Ermilov & Anichkin, 2014 [Ermilov and Anichkin 2014d]

Novosuctobelba (Novosuctobelba) vietnamica Balogh & Mahunka, 1967 [Balogh and Mahunka 1967]

*Suctobelbata bituberculata* Ermilov & Anichkin, 2013 [Ermilov and Anichkin 2013c]

Suctobelbella (Suctobelbella) elegantissima (Hammer, 1979) [Golosova 1983]

Suctobelbella (Suctobelbella) finlayi (Balogh & Mahunka, 1980) [Nguyen and Vu 2012]

Suctobelbella (Suctobelbella) latirostris (Strenzke, 1950) [Vu 1990]

Suctobelbella (Suctobelbella) longicuspis Jacot, 1937 [Nguyen and Vu 2012]

Suctobelbella (Flagrosuctobelba) elegantula (Hammer, 1958) [Ermilov et al. 2012c]

Suctobelbella (Flagrosuctobelba) parallelodentata Hammer, 1979 [Ermilov et al. 2012c]

Suctobelbella (Flagrosuctobelba) semiplumosa (Balogh & Mahunka, 1967) [Balogh and Mahunka 1967]

Suctobelbella (Flagrosuctobelba) subtrigona (Oudemans, 1900) [Vu et al. 2012]

Suctobelbella (Ussuribata) bivittata (Hammer, 1979) [Ermilov and Anichkin 2014d]

Suctobelbella (Ussuribata) multituberculata Balogh & Mahunka, 1967 [Balogh and Mahunka 1967]

Suctobelbella (Ussuribata) phylliformis Ermilov, Shtanchaeva & Subías, 2014 [Ermilov et al. 2014d]

Suctobelbella (Ussuribata) sexsetosa (Hammer, 1979) [Ermilov and Anichkin 2013d]

Suctobelbella (Ussuribata) variosetosa (Hammer, 1961) [Golosova 1983]

*Suctobelbila minima* Hammer, 1979 [Golosova 1983]

*Suctobelbila multituberculata* Hammer, 1979 [Ermilov et al. 2012c]

*Suctobelbila scutata* Hammer, 1972 [Ermilov and Anichkin 2014d]

*Suctobelbila transrugosa* (Mahunka, 1986) [Nguyen and Vu 2012]

**Oxyameridae**

*Oxyamerus aokii* Balogh, 1968 [Ermilov and Anichkin 2014d]

*Oxyamerus hyalinus* Hammer, 1979 [Ermilov and Anichkin 2014d]

*Oxyamerus truncatus* Hammer, 1979 [Ermilov and Anichkin 2014d]

**Dampfiellidae**

*Dampfiella angusta* Hammer, 1979 [Ermilov and Anichkin 2014d]

**Otocepheidae**

*Basiceramerus igorotus* Corpuz-Raros and Gruèzo, 2011 [Ermilov and Anichkin 2013g]

*Eurostocepheus aquilinus* Aoki, 1965 [Ermilov et al. 2012c]

*Dolicheremaeus aokii* Balogh & Mahunka, 1967 [Balogh and Mahunka 1967]

*Dolicheremaeus baloghi* Aoki, 1967 [Ermilov and Bayartogtokh 2005]

*Dolicheremaeus bartkei* Rajski & Szudrowicz, 1974 [Rajski and Szudrowicz 1974]

*Dolicheremaeus bruneiensis* Aoki, 1967 [Ermilov and Bayartogtokh 2005]

*Dolicheremaeus bugiamapensis* Ermilov, Anichkin & Wu, 2012 [Ermilov et al. 2012a]

*Dolicheremaeus capillatus* (Balogh, 1959) [Nguyen and Vu 2012]

*Dolicheremaeus contactus* Ermilov & Anichkin, 2013 [Ermilov and Anichkin 2013d]

*Dolicheremaeus damaeoides* (Berlese, 1913) [Ermilov and Anichkin 2014d]

*Dolicheremaeus donacunarensis* Ermilov & Anichkin, 2014 [Ermilov and Anichkin 2014g]

*Dolicheremaeus dwalteri* Ermilov & Anichkin, 2014 [Ermilov and Anichkin 2014g]

*Dolicheremaeus insolitus* Ermilov & Anichkin, 2014 [Ermilov and Anichkin 2014d]

*Dolicheremaeus junichiaokii* Subías, 2010 (=*Dolicheremaeus magnus* Aoki, 2006) [Ermilov and Anichkin 2013g]

*Dolicheremaeus inaequalis* Balogh & Mahunka, 1967 [Balogh and Mahunka 1967]

*Dolicheremaeus lineolatus* Balogh & Mahunka, 1967 [Balogh and Mahunka 1967]

*Dolicheremaeus orientalis* (Aoki, 1965) [Vu 1994]

*Dolicheremaeus ornatus* Balogh & Mahunka, 1967 [Balogh and Mahunka 1967]

*Dolicheremaeus philippinensis* Aoki, 1967 [Vu et al. 2014]

*Dolicheremaeus sabahnus* Mahunka, 1988 [Nguyen and Vu 2012]

Fissicepheus (Fissicepheus) elegans Balogh & Mahunka, 1967 [Balogh and Mahunka 1967]

Fissicepheus (Fissicepheus) striganovae Ermilov & Anichkin, 2014 [Ermilov and Anichkin 2014d]

*Leptotocepheus murphyi* (Mahunka, 1989) [Ermilov and Bayartogtokh 2015]

Megalotocepheus (Archegotocepheus) crinitus Berlese, 1905 [Ermilov and Anichkin 2014d]

Megalotocepheus (Archegotocepheus) singularis (Mahunka, 1988) [Nguyen and Vu 2012]

Otocepheus (Otocepheus) spatulatus Mahunka, 2000 [Ermilov and Anichkin 2013c]

Otocepheus (Acrotocepheus) duplicornutus duplicornutus Aoki, 1965 [Vu and Nguyen 2005]

Otocepheus (Acrotocepheus) duplicornutus discrepans Balogh & Mahunka, 1967 [Balogh and Mahunka 1967]

Otocepheus (Acrotocepheus) excelsus Aoki, 1965 [Ermilov and Anichkin 2013d]

Otocepheus (Acrotocepheus) triplicicornutus Balogh & Mahunka, 1967 [Balogh and Mahunka 1967]

Otocepheus (Acrotocepheus) vietnamicus Ermilov & Anichkin, 2011 [Ermilov and Anichkin 2011e]

*Papillocepheus primus* Ermilov, Anichkin & Tolstikov, 2014 [Ermilov et al. 2014b]

*Pseudotocepheus setiger* (Hammer, 1972) [Ermilov et al. 2013a]

*Umashtanchaeviella plethotricha* Ermilov, Anichkin & Tolstikov, 2014 [Ermilov et al. 2014c]

**Carabodidae**

*Aokiella florens* Balogh & Mahunka, 1967 [Balogh and Mahunka 1967]

*Aokiella rotunda* Hammer, 1979 [Ermilov et al. 2012b]

*Aokiella xuansoni* Vu, Ermilov & Dao, 2010 [Vu et al. 2010]

Austrocarabodes (Austrocarabodes) alveolatus Hammer, 1973 [Vu et al. 2014]

Austrocarabodes (Austrocarabodes) falcatus Hammer, 1973 [Nguyen and Vu 2012]

Austrocarabodes (Austrocarabodes) szentivanyi (Balogh & Mahunka, 1967) [Balogh and Mahunka 1967]

Austrocarabodes (Austrocarabodes) vaucheri Mahunka, 1984 [Nguyen and Vu 2012]

Austrocarabodes (Uluguroides) polytrichus Balogh & Mahunka, 1978 [Vu et al. 2015]

Carabodes (Klapperiches) mikhaetandreorum Ermilov & Anichkin, 2013 [Ermilov and Anichkin 2013g]

Carabodes (Klapperiches) samoensis Balogh & Balogh, 1986 [Ermilov and Anichkin 2013d]

Carabodes (Klapperiches) strinovichi Balogh & Mahunka, 1978 [Vu et al. 2014]

Carabodes (Phyllocarabodes) inopinatus (Mahunka, 1985) [Nguyen and Vu 2012]

Carabodes (Phyllocarabodes) schatzi Subías, 2010 [as *Phyllocarabodes ornatus* Balogh, 1986 – Nguyen and Vu 2012]

*Chistyakovella insolita* Ermilov, Aoki & Anichkin, 2013 [Ermilov et al. 2013c]

Gibbicepheus (Gibbicepheus) baccanensis Jeleva & Vu, 1987 [Jeleva and Vu 1987]

Gibbicepheus (Gibbicepheus) fenestralis Hammer, 1979 [Golosova 1983]

Gibbicepheus (Gibbicepheus) latohumeralis Hammer, 1982 [Ermilov and Anichkin 2015a]

Yoshiobodes (Yoshiobodes) irmayi (Balogh & Mahunka, 1969) [as *Yoshiobodes aokii* Mahunka, 1987 – Ermilov and Anichkin 2013c]

Yoshiobodes (Yoshiobodes) neotrichorostralis Ermilov, Shtanchaeva, Subías & Anichkin, 2014 [Ermilov et al. 2014e]

Yoshiobodes (Dongnaibodes) biconcavus Ermilov, Shtanchaeva, Subías & Anichkin, 2014 [Ermilov et al. 2014e]

Yoshiobodes (Dongnaibodes) hexasetosus Ermilov, Shtanchaeva, Subías & Anichkin, 2014 [Ermilov et al. 2014e]

**Nippobodidae**

*Nippobodes monstruosus* (Jeleva & Vu, 1987) [Jeleva and Vu 1987]

**Tectocepheidae**

*Tectocepheus elegans* Ohkubo, 1981 [Vu et al. 2014]

*Tectocepheus minor* Berlese, 1903 [as *Tectocepheus cuspidentatus* Knülle, 1954 – Vu 1993]

*Tectocepheus velatus* (Michael, 1880) [Golosova 1983]

*Tegeozetes tunicatus tunicatus* Berlese, 1913 [Ermilov and Anichkin 2013f]

*Tegeozetes tunicatus breviclava* Aoki, 1970 [Vu 2013]

**Tegeocranellidae**

*Tegeocranellus martinezi* Ermilov & Anichkin, 2014 [Ermilov and Anichkin 2014d]

**Microtegeidae**

*Microtegeus borhidii* Balogh & Mahunka, 1974 [Ermilov et al. 2012a]

*Microtegeus cardosensis* Pérez-Íñigo, 1985 [Vu et al. 2014]

*Microtegeus cornutus* Balogh, 1970 [Nguyen and Vu 2012]

*Microtegeus quadristriatus* Mahunka, 1984 [Nguyen and Vu 2012]

*Microtegeus reticulatus* Aoki, 1965 [Vu 1990]

**Cymbaeremaeidae**

*Scapheremaeus ascissuratus* Ermilov & Anichkin, 2015 [Ermilov and Anichkin 2015b]

*Scapheremaeus cellulatifer* Mahunka, 1987 [Mahunka 1987]

*Scapheremaeus crassus* Mahunka, 1988 [Mahunka 1988]

*Scapheremaeus fisheri* Aoki, 1966 [Ermilov et al. 2012c]

*Scapheremaeus foveolatus* Mahunka, 1987 [Mahunka 1987]

**Licneremaeidae**

*Licneremaeus licnophorus* (Michael, 1882) [Vu et al. 2014]

*Licneremaeus polygonalis* Hammer, 1971 [Ermilov et al. 2013a]

**Phenopelopidae**

*Eupelops forsslundi* (Balogh, 1959) [Vu et al. 1987]

*Nesopelops intermedius* Hammer, 1979 [Ermilov and Anichkin 2013g]

**Eremaeozetidae**

*Mahunkaia bituberculata* (Mahunka, 1983) [Nguyen and Vu 2012]

**Idiozetidae**

*Idiozetes javensis* Hammer, 1979 [Ermilov et al. 2012a]

**Limnozetidae**

*Limnozetes pustulatus* (Mahunka, 1987) [Mahunka 1987]

**Microzetidae**

*Berlesezetes ornatissimus* (Berlese, 1913) [as *Berlesezetes auxiliaris* Grandjean, 1936 – Mahunka 1988]

*Caucasiozetes frankeae* Ermilov & Anichkin, 2011 [Ermilov and Anichkin 2011e]

*Kaszabodes velatus* Mahunka, 1988 [Mahunka 1988]

*Schalleriella vietnamica* Ermilov & Anichkin, 2011 [Ermilov and Anichkin 2011a]

**Achipteriidae**

Achipteria (Achipteria) curta Aoki, 1970 [Vu 1993]

Anachipteria (Anachipteria) svetlanae Ermilov & Anichkin, 2014 [Ermilov and Anichkin 2014d]

*Austrachipteria phongnhae* Ermilov & Vu, 2012 [Ermilov and Vu 2012]

*Campachipteria distincta* (Aoki, 1959) [Vu and Nguyen 2000]

*Campachipteria uenoi* Aoki, 1995 [Ermilov and Anichkin 2014d]

*Plakoribates asiaticus* Ermilov & Anichkin, 2013 [Ermilov and Anichkin 2013a]

**Tegoribatidae**

*Ceratobates cangioensis* Ermilov & Anichkin, 2015 [Ermilov and Anichkin 2015a]

*Tegoribates americanus* Hammer, 1958 [Vu et al. 2014]

**Oribatellidae**

*Novoribatella minutisetarum* Engelbrecht, 1986 [Nguyen and Vu 2012]

*Ophidiotrichus ussuricus* Krivolutsky, 1971 [Vu et al. 2014]

Oribatella (Oribatella) gerdweigmanni Ermilov & Anichkin, 2012 [Ermilov and Anichkin 2012d]

Oribatella (Oribatella) illuminata Hammer, 1961 [Vu et al. 2014]

Oribatella (Oribatella) prolongata Hammer, 1961 [Vu et al. 2014]

Oribatella (Oribatella) sculpturata Mahunka, 1987 [Mahunka 1987]

Oribatella (Oribatella) umaetluisorum Ermilov & Anichkin, 2012 [Ermilov and Anichkin 2012a]

**Heterozetidae**

*Farchacarus philippinensis* (Corpuz-Raros, 1979) [Golosova 1983]

**Ceratozetidae**

Ceratozetes (Ceratozetes) bicornis Hammer, 1967 [Golosova 1983]

Ceratozetes (Ceratozetes) gracilis (Michael, 1884) [Golosova 1983]

Ceratozetes (Ceratozetes) mediocris Berlese, 1908 [Golosova 1983]

*Fuscozetes fuscipes* (Koch, 1844) [Vu 1990]

*Lepidozetes trifolius* (Fujikawa, 1972) [Vu et al. 2014]

*Sphaerozetes bugiamapensis* Ermilov, Anichkin & Wu, 2013 [Ermilov et al. 2013b]

**Punctoribatidae**

*Allozetes africanus* Balogh, 1958 [Golosova 1983]

*Allozetes pusillus* (Berlese, 1913) [Mahunka 1988]

*Lamellobates molecula* (Berlese, 1916) [Golosova 1983; also as *Lamellobates palustris* Hammer, 1958 – Vu et al. 1985; as *Lamellobates hauseri* Mahunka, 1977 – Mahunka 1988]

*Lamellobates ocularis* Jeleva & Vu, 1987 [Jeleva and Vu 1987]

*Paralamellobates misella* (Berlese, 1910) [as *Paralamellobates schoutedani* (Balogh, 1959) – Vu et al. 1985; also as *Paralamellobates ceylanicus* (Oudemans, 1916) – Mahunka 1988]

*Punctoribates hexagonus* Berlese, 1908 [Vu 1990]

*Punctoribates punctum* (Koch, 1839) [Pavlichenko 1991]

**Chamobatidae**

Chamobates (Chamobates) javensis (Hammer, 1979) [Golosova 1983]

**Mochlozetidae**

*Mochlozetes ryukyuensis* Aoki, 2006 [Ermilov and Anichkin 2013d]

*Unguizetes asiaticus* Ermilov & Anichkin, 2012 [Ermilov and Anichkin 2012b]

*Unguizetes cattienensis* Ermilov & Anichkin, 2011 [Ermilov and Anichkin 2011e]

*Unguizetes clavatus* Aoki, 1967 [Golosova 1983]

*Unguizetes latus* Ermilov & Anichkin, 2013 [Ermilov and Anichkin 2013d]

*Unguizetes sphaerula* (Berlese, 1905) [Ermilov et al. 2012c]

Uracrobates (Uracrobates) magniporosus Balogh & Mahunka, 1967 [Balogh and Mahunka 1967]

**Oribatulidae**

*Paraphauloppia gracilis* (Hammer, 1958) [Nguyen and Vu 2012]

*Zygoribatula pennata* Grobler, 1993 [Nguyen and Vu 2012]

*Zygoribatula prima* Ermilov & Anichkin, 2011 [Ermilov and Anichkin 2011k]

*Zygoribatula undulata* Berlese, 1916 [as *Zygoribatula longiporosa* Hammer, 1953 – Vu et al. 2012]

**Sellnickiidae**

*Sellnickia caudata* (Michael, 1908) [Ermilov et al. 2012c]

**Caloppiidae**

*Zetorchella latior* (Berlese, 1913) [Ermilov et al. 2012c]

*Zetorchella reticulata* (Willmann, 1933) [Ermilov and Anichkin 2013g]

**Scheloribatidae**

*Areozetes incertus* Balogh, 1970 [Vu et al. 2014]

*Cordiozetes olahi* (Mahunka, 1987) [Mahunka 1987]

Euscheloribates (Euscheloribates) samsinaki Kunst, 1958 [Vu 1990]

Euscheloribates (Trischeloribates) clavatus (Mahunka, 1988) [Mahunka 1988]

Exoribatula (Multoribates) longior (Hammer, 1958) [Ermilov and Anichkin 2014d]

*Fijibates aelleni* (Mahunka, 1988) [Ermilov et al. 2012c]

*Fijibates rostratus* Hammer, 1971 [Ermilov et al. 2013a]

Liebstadia (Liebstadia) humerata Sellnick, 1928 [Vu 1993]

Perscheloribates (Perscheloribates) lanceolatus (Aoki, 1984) [Vu et al. 2008]

Perscheloribates (Perscheloribates) luminosus (Hammer, 1961) [Ermilov and Anichkin 2014d]

Perscheloribates (Perscheloribates) luteus (Hammer, 1962) [Nguyen and Vu 2012]

Perscheloribates (Perscheloribates) minutus (Pletzen, 1965) [Golosova 1983]

*Rhabdoribates siamensis* Aoki, 1967 [Vu 1990]

Scheloribates (Scheloribates) crucisetus Jeleva & Vu, 1987 [Jeleva and Vu 1987]

Scheloribates (Scheloribates) fimbriatus Thor, 1930 [Vu et al. 1985]

Scheloribates (Scheloribates) flagellisetosus Ermilov & Anichkin, 2014 [Ermilov and Anichkin 2014c]

Scheloribates (Scheloribates) kraepelini (Berlese, 1908) [Ermilov and Anichkin 2013e]

Scheloribates (Scheloribates) laevigatus (Koch, 1835) [Vu et al. 1985]

Scheloribates (Scheloribates) latipes (Koch, 1844) [Golosova 1983]

Scheloribates (Scheloribates) pallidulus (Koch, 1844) [Vu et al. 1985]

Scheloribates (Scheloribates) parvus Pletzen, 1963 [Vu et al. 2015]

Scheloribates (Scheloribates) praeincisus praeincisus (Berlese, 1910) [Golosova 1983]

Scheloribates (Scheloribates) praeincisus interruptus (Berlese, 1916) [Ermilov and Anichkin 2013g]

Scheloribates (Scheloribates) vulgaris Hammer, 1961 [Nguyen and Vu 2012]

Scheloribates (Bischeloribates) mahunkai Subías, 2010 [as *Philoribates heterodactylus* (Mahunka, 1988) – Nguyen and Vu 2012]

*Tuberemaeus lineatus* Balogh, 1970 [Nguyen and Vu 2012]

*Tuberemaeus perforatoides* Hammer, 1979 [Ermilov and Anichkin 2013d]

*Tuberemaeus sculpturatus* Mahunka, 1987 [Mahunka 1987]

*Tuberemaeus singularis* Sellnick, 1930 [Ermilov and Anichkin 2013g]

*Vesiculobates silvaticus* Hammer, 1979 [Ermilov and Anichkin 2014f]

**Oripodidae**

*Brachyoripoda foveolata* Balogh, 1970 [Ermilov et al. 2012c]

*Cosmopirnodus tridactylus* Mahunka, 1988 [Mahunka 1988]

*Oripoda excavata* Mahunka, 1988 [Mahunka 1988]

*Oripoda pinicola* Aoki & Ohkubo, 1974 [Vu et al. 2008]

*Subpirnodus mirabilis* Mahunka, 1988 [Mahunka 1988]

*Truncopes moderatus variabilis* Aoki & Yamamoto, 2007 [Ermilov and Anichkin 2013f]

*Truncopes orientalis* Mahunka, 1987 [Mahunka 1987]

**Haplozetidae**

*Acutozetes rostratus* Balogh, 1970 [Vu et al. 2014]

Indoribates (Indoribates) bicarinatus Ermilov & Anichkin, 2014 [Ermilov and Anichkin 2014d]

Indoribates (Indoribates) microsetosus Ermilov & Anichkin, 2011 [Ermilov and Anichkin 2011j]

Indoribates (Indoribates) nobilis (Golosova, 1984) [Golosova 1984]

Indoribates (Indoribates) punctulatus (Sellnick, 1925) [as *Indoribates panabokkei* (Balogh, 1970) – Ermilov and Anichkin 2013e]

*Haplozetes vindobonensis* (Willmann, 1935) [Ermilov et al. 2012c]

Lauritzenia (Incabates) major (Aoki, 1967) [Vu et al. 2014]

Lauritzenia (Magnobates) glagellifer (Hammer, 1967) [Vu 1990]

Peloribates (Peloribates) gressitti Balogh & Mahunka, 1967 [Balogh and Mahunka 1967]

Peloribates (Peloribates) guttatoides Hammer, 1979 [Ermilov and Anichkin 2014f]

Peloribates (Peloribates) guttatus Hammer, 1979 [Vu et al. 2014]

Peloribates (Peloribates) kaszabi Mahunka, 1988 [Mahunka 1988]

Peloribates (Peloribates) paraguayensis Balogh & Mahunka, 1981 [Nguyen and Vu 2012]

Peloribates (Peloribates) pseudoporosus Balogh & Mahunka, 1967 [Balogh and Mahunka 1967]

Peloribates (Peloribates) rangiroaensis Hammer, 1972 [Ermilov and Anichkin 2011k]

Peloribates (Peloribates) ratubakensis Hammer, 1979 [Vu et al. 2014]

Peloribates (Peloribates) spiniformis Ermilov & Anichkin, 2011 [Ermilov and Anichkin 2011k]

Peloribates (Peloribates) stellatus Balogh & Mahunka, 1967 [Balogh and Mahunka 1967]

Peloribates (Peloribates) tatyanae Ermilov & Anichkin, 2014 [Ermilov and Anichkin 2014d]

*Perxylobates brevisetosus* Mahunka, 1988 [Mahunka 1988]

*Perxylobates crassisetosus* Ermilov & Anichkin, 2011 [Ermilov and Anichkin 2011j]

*Perxylobates guehoi* Mahunka, 1978 [Nguyen and Vu 2012]

*Perxylobates thanhoaensis* Ermilov, Vu, Trinh & Dao, 2011 [Ermilov et al. 2011c]

*Perxylobates vermisetus* (Balogh & Mahunka, 1968) [Vu et al. 1985]

*Perxylobates vietnamensis* (Jeleva & Vu, 1987) [Jeleva and Vu 1987]

Protoribates (Protoribates) capucinus Berlese, 1908 [Golosova 1983]

Protoribates (Protoribates) cattienensis Ermilov & Anichkin, 2011 [Ermilov and Anichkin 2011j]

Protoribates (Protoribates) dentatus (Berlese, 1883) [as *Protoribates monodactylus* (Haller, 1884) – Vu et al. 1985]

Protoribates (Protoribates) gracilis (Aoki, 1982) [Vu and Nguyen 2000]

Protoribates (Protoribates) lophotrichus (Berlese, 1904) [Vu et al. 1985]

Protoribates (Protoribates) paracapucinus (Mahunka, 1988) [Ermilov et al. 2012c]

Protoribates (Triaunguis) acutus (Hammer, 1979) [Golosova 1983]

Protoribates (Triaunguis) bisculpturatus (Mahunka, 1988) [Vu et al. 2014]

Protoribates (Triaunguis) duoseta (Hammer, 1979) [Nguyen and Vu 2012]

Protoribates (Triaunguis) heterodactylus Ermilov & Anichkin, 2011 [Ermilov and Anichkin 2011c]

Protoribates (Triaunguis) maximus (Mahunka, 1988) [Mahunka 1988]

Setoxylobates (Setoxylobates) foveolatus Balogh & Mahunka, 1967 [Balogh and Mahunka 1967]

Setoxylobates (Polyxylobates) diversiporosus (Hammer, 1973) [Ermilov and Anichkin 2015a]

*Trachyoribates irregularis* (Balogh & Mahunka, 1969) [Vu et al. 1985]

*Trachyoribates ovulum* Berlese, 1908 [as *Rostrozetes foveolatus* Sellnick, 1925 – Golosova 1983; also as *Rostrozetes areolatus* (Balogh, 1958) – Vu et al. 1985; as *Rostrozetes punctulifer* Balogh & Mahunka, 1979 – Vu et al. 1987; as *Rostrozetes trimorphus* Balogh & Mahunka, 1979 – Vu 1990]

*Transoribates agricola* (Nakamura & Aoki, 1989) [Ermilov and Anichkin 2014f]

*Vilhenabates sinatus* (Aoki, 1965) [Ermilov et al. 2012a]

**Parakalummidae**

Neoribates (Neoribates) aurantiacus (Oudemans, 1914) [Vu 1990]

Neoribates (Neoribates) jacoti (Balogh & Mahunka, 1967) [Balogh and Mahunka 1967]

Neoribates (Neoribates) monodactylus Ermilov & Anichkin, 2014 [Ermilov and Anichkin 2014d]

Neoribates (Neoribates) paratuberculatus Ermilov, Shtanchaeva & Subías, 2014 [Ermilov et al. 2014d]

Neoribates (Neoribates) spindleformis Ermilov & Anichkin, 2012 [Ermilov and Anichkin 2012c]

**Galumnidae**

Allogalumna (Allogalumna) bipartita (Aoki & Hu, 1993) [Ermilov and Anichkin 2013c]

Allogalumna (Allogalumna) costata Mahunka, 1996 [Vu et al. 2014]

Allogalumna (Allogalumna) monodactyla Ermilov & Anichkin, 2014 [Ermilov and Anichkin 2014e]

Allogalumna (Allogalumna) multesima Grandjean, 1957 [Nguyen and Vu 2012]

Allogalumna (Allogalumna) paramachadoi Ermilov & Anichkin, 2014 [Ermilov and Anichkin 2014d]

Allogalumna (Allogalumna) rotundiceps Aoki, 1996 [Ermilov and Anichkin 2014d]

Allogalumna (Allogalumna) upoluensis Hammer, 1973 [Vu et al. 2014]

Allogalumna (Globogalumna) biporosa Ermilov & Anichkin, 2012 [Ermilov and Anichkin 2012c]

*Dimidiogalumna azumai* Aoki, 1996 [Nguyen et al. 2013]

*Dimidiogalumna grandjeani* Ermilov & Anichkin, 2014 [Ermilov and Anichkin 2014a]

Galumna (Galumna) aba Mahunka, 1989 [Mahunka 1989]

Galumna (Galumna) acutirostrum Ermilov & Anichkin, 2010 [Ermilov and Anichkin 2010]

Galumna (Galumna) coronata Mahunka, 1992 [Vu et al. 2015]

Galumna (Galumna) discifera Balogh, 1960 [Nguyen and Vu 2012]

Galumna (Galumna) flabellifera Hammer, 1958 [as *Galumna flabellifera orientalis* Aoki, 1965 – Vu 1990]

Galumna (Galumna) kebangica Ermilov & Vu, 2012 [Ermilov and Vu 2012]

Galumna (Galumna) khoii Mahunka, 1989 [Mahunka 1989]

Galumna (Galumna) lanceata (Oudemans, 1900) [Golosova 1983]

Galumna (Galumna) levisensilla Ermilov & Anichkin, 2010 [Ermilov and Anichkin 2010]

Galumna (Galumna) microfissum Hammer, 1968 [Vu et al. 2014]

Galumna (Galumna) obvia (Berlese, 1914) [Vu 1993]

Galumna (Galumna) paracalcicola Ermilov & Anichkin, 2014 [Ermilov and Anichkin 2014e]

Galumna (Galumna) parakazakhstani Ermilov & Anichkin, 2014 [Ermilov and Anichkin 2014b]

Galumna (Galumna) paramastigophora Ermilov, 2015 [Ermilov 2015a]

Galumna (Galumna) pseudokhoii Ermilov & Anichkin, 2011 [Ermilov and Anichkin 2011h]

Galumna (Galumna) pseudotriquetra Ermilov, 2015 [Ermilov 2015a]

Galumna (Galumna) triquetra Aoki, 1965 [Vu 1990]

Galumna (Galumna) triops Balogh, 1960 [Vu et al. 2015]

Galumna (Cosmogalumna) dongnaiensis Ermilov & Anichkin, 2013 [Ermilov and Anichkin 2013f]

Galumna (Cosmogalumna) praeoccupata Subías, 2004 [Ermilov and Anichkin 2014d]

Galumna (Cosmogalumna) tenensis Ermilov, Vu & Nguyen, 2011 [Ermilov et al. 2011b]

Galumna (Neogalumna) longilineata Ermilov & Anichkin, 2014 [Ermilov and Anichkin 2014d]

Galumna (Neogalumna) seniczaki Ermilov & Anichkin, 2010 [Ermilov and Anichkin 2010]

Galumna (Neogalumna) tolstikovi Ermilov & Anichkin, 2014 [Ermilov and Anichkin 2014d]

Leptogalumna (Leptogalumna) ciliata Balogh, 1960 [Ermilov and Anichkin 2011h]

*Notogalumna foveolata* Balakrishnan, 1989 [Ermilov and Anichkin 2013g]

*Notogalumna lagunaensis* Ermilov & Corpuz-Raros, 2015 [Ermilov and Corpuz-Raros 2015]

Pergalumna (Pergalumna) altera (Oudemans, 1915) [Vu et al. 1985]

Pergalumna (Pergalumna) cattienica Ermilov & Anichkin, 2011 [Ermilov and Anichkin 2011f]

Pergalumna (Pergalumna) granulata Balogh & Mahunka, 1967 [Balogh and Mahunka 1967]

Pergalumna (Pergalumna) hauseri Mahunka, 1995 [Ermilov and Anichkin 2013d]

Pergalumna (Pergalumna) indistincta Ermilov & Anichkin, 2011 [Ermilov and Anichkin 2011h]

Pergalumna (Pergalumna) kotschyi Mahunka, 1989 [Mahunka 1989]

Pergalumna (Pergalumna) longisetosa Balogh, 1960 [Vu et al. 2014]

Pergalumna (Pergalumna) magnipora capillaris Aoki, 1961 [Vu 1993]

Pergalumna (Pergalumna) margaritata Mahunka, 1989 [Mahunka 1989]

Pergalumna (Pergalumna) mauritii Mahunka, 1978 [Vu et al. 2014]

Pergalumna (Pergalumna) montana Hammer, 1961 [Vu et al. 2015]

Pergalumna (Pergalumna) nuda Balogh, 1960 [Vu et al. 2014]

Pergalumna (Pergalumna) paraelongata Ermilov & Anichkin, 2012 [Ermilov et al. 2012c]

Pergalumna (Pergalumna) pseudosejugalis Ermilov & Anichkin, 2012 [Ermilov and Anichkin 2012d]

Pergalumna (Pergalumna) punctulata Balogh & Mahunka, 1967 [Balogh and Mahunka 1967]

Pergalumna (Pergalumna) taprobanica Balogh, 1988 [Vu et al. 2014]

Pergalumna (Pergalumna) yurtaevi Ermilov & Anichkin, 2011 [Ermilov and Anichkin 2011f]

*Trichogalumna nipponica* (Aoki, 1966) [Ermilov et al. 2012c]

*Trichogalumna subnuda* Balogh & Mahunka, 1967 [Balogh and Mahunka 1967]

*Trichogalumna vietnamica* Mahunka, 1987 [Mahunka 1987]

**Galumnellidae**

Galumnella (Galumnella) cellularis (Balogh & Mahunka, 1967) [Balogh and Mahunka 1967]

Galumnella (Galumnella) geographica Mahunka, 1995 [Vu et al. 2014]

Galumnella (Galumnella) microporosa Ermilov & Anichkin, 2011 [Ermilov and Anichkin 2011f]

Galumnella (Galumnella) tiunovi Ermilov & Anichkin, 2013 [Ermilov and Anichkin 2013d]

Galumnella (Galumnella) paulinai Balogh, 1961 [Vu et al. 2014]

Galumnella (Bagalumnella) scavasorum (Mahunka, 1994) [Vu et al. 2014]

*Porogalumnella pulchella* Aoki & Hu, 1993 [Ermilov and Anichkin 2014d]

## Conclusion

The list of oribatid mites of Vietnam includes now 535 species/subspecies, 222 genera and 81 families. Of these, 194 species/subspecies were described as new for science from Vietnam; 94 species have been described by Ermilov and co-authors, 30 species by Balogh and Mahunka, 28 species by Mahunka, 21 species by Niedbała, 6 species by Jeleva and Vu, 4 species by Fernandez and co-authors, 3 species by Krivolutsky, 2 species by Golosova, 2 species by Starý, 2 species by Vu and co-authors, 1 species by Balogh, 1 species by Rajski and Szudrowicz.
